# Persistent Feeding and Swallowing Deficits in a Mouse Model of 22q11.2 Deletion Syndrome

**DOI:** 10.3389/fneur.2020.00004

**Published:** 2020-01-31

**Authors:** Lauren Welby, Hailey Caudill, Gelila Yitsege, Ali Hamad, Filiz Bunyak, Irene E. Zohn, Thomas Maynard, Anthony-Samuel LaMantia, David Mendelowitz, Teresa E. Lever

**Affiliations:** ^1^Department of Otolaryngology Head and Neck Surgery, School of Medicine, University of Missouri, Columbia, MO, United States; ^2^Department of Pharmacology and Physiology, School of Medicine and Health Sciences, The George Washington University, Washington, DC, United States; ^3^Center for Genetic Medicine and the Center for Neuroscience Research, Children's National Medical Center, Children's National Health Systems, Washington, DC, United States; ^4^School of Medicine and Health Sciences, Institute for Neuroscience, The George Washington University, Washington, DC, United States; ^5^Department of Electrical Engineering and Computer Science, University of Missouri, Columbia, MO, United States; ^6^Departments of Pediatrics, School of Medicine and Health Sciences, The George Washington University, Washington, DC, United States; ^7^Laboratory of Developmental Disorders and Genetics, Virginia Tech-Carilion School of Medicine, The Fralin Biomedical Research Institute, Roanoke, VA, United States; ^8^Department of Biology, Virginia Tech, Blacksburg, VA, United States

**Keywords:** 22q11 deletion syndrome, DiGeorge syndrome, pediatric dysphagia, dysphagia, deglutition, feeding, mouse model

## Abstract

Disrupted development of oropharyngeal structures as well as cranial nerve and brainstem circuits may lead to feeding and swallowing difficulties in children with 22q11. 2 deletion syndrome (22q11DS). We previously demonstrated aspiration-based dysphagia during early postnatal life in the *LgDel* mouse model of 22q11DS along with disrupted oropharyngeal morphogenesis and divergent differentiation and function of cranial motor and sensory nerves. We now ask whether feeding and swallowing deficits persist in adult *LgDel* mice using methods analogous to those used in human patients to evaluate feeding and swallowing dysfunction. Compared to wild-type mice, videofluoroscopic swallow study revealed that *LgDel* mice have altered feeding and swallowing behaviors, including slower lick rates, longer inter-lick intervals, and longer pharyngeal transit times with liquid consistency. Transoral endoscopic assessment identified minor structural anomalies of the palate and larynx in one-third of the *LgDel* mice examined. Video surveillance of feeding-related behaviors showed that *LgDel* mice eat and drink more frequently. Furthermore, *LgDel* animals engage in another oromotor behavior, grooming, more frequently, implying that divergent craniofacial and cranial nerve structure and function result in altered oromotor coordination. Finally, *LgDel* mice have significantly increased lung inflammation, a potential sign of aspiration-based dysphagia, consistent with results from our previous studies of early postnatal animals showing aspiration-related lung inflammation. Thus, oromotor dysfunction, feeding, and swallowing difficulties and their consequences persist in the *LgDel* 22q11DS mouse model. Apparently, postnatal growth and/or neural plasticity does not fully resolve deficits due to anomalous hindbrain, craniofacial, and cranial nerve development that prefigure perinatal dysphagia in 22q11DS. This new recognition of persistent challenges with feeding and swallowing may provide opportunities for improved therapeutic intervention for adolescents and adults with 22q11DS, as well as others with a history of perinatal feeding and swallowing disorders.

## Introduction

Almost all infants with 22q11.2 Deletion Syndrome (22q11DS) have pediatric dysphagia—perinatal difficulties with suckling, feeding, and swallowing (1). As a consequence, many children with 22q11DS have recurrent naso-sinus and respiratory infections, impaired speech development, and failure to thrive (2, 3). Clinically significant dysphagia continues in approximately one-third of individuals with 22q11DS as they mature, and approximately half will require enteral feeding interventions (1). Our previous work demonstrates that newborn *LgDel* mice—a genomically accurate 22q11DS model that carries a heterozygous deletion of 28 contiguous genes on mouse chromosome 16, orthologous to the minimal 1.5 MB critical region on human chromosome 22 deleted in 22q11DS (4, 5)—exhibit multiple signs of pediatric dysphagia (6, 7). It is not clear, however, whether maturation or compensatory changes including neural circuit plasticity correct or at least diminish presumed developmental pathology. Thus, we asked whether dysphagic symptoms continue into maturity in adult *LgDel* mice using high resolution video and fluorographic analysis of oromotor function and feeding-related behaviors.

Several clinically significant 22q11DS phenotypes, including pediatric dysphagia, emerge during infancy and early life (2, 8–10). Many of these phenotypes reflect disruptions of the developmental program for embryonic pharyngeal morphogenesis (11). Nevertheless, feeding difficulties in 22q11DS are apparently independent of palatal and/or cardiac disruption and instead reflect poor coordination of the suck/swallow/breathing pattern (1), implicating altered neural circuit differentiation in this 22q11DS clinical complication. Disrupted patterning of the embryonic hindbrain, as well as divergent development of cranial nerves (CNs) V, IX, and X precede these anomalies (7). Despite these developmentally established differences, it remains unclear whether apparently related perinatal feeding and swallowing difficulties are mostly resolved subsequently, or whether they persist, introducing ongoing challenges for essential oromotor behaviors throughout life.

Accordingly, we characterized feeding and swallowing related behaviors as well as oropharyngeal and craniofacial morphology in adult *LgDel* mice and wild type (WT) controls and assessed additional signs of aspiration-related swallowing difficulties. We assessed functional phenotypes related to dysphagia using fluoroscopic and endoscopic approaches as well as automated video-based monitoring and computational analysis of baseline feeding behaviors. We found that *LgDel* adult mice have persistent oromotor control difficulties, disrupted feeding, and aspiration-related lung inflammation. These studies establish methods for continued analysis of the consequences of underlying developmental origins of dysphagia and a preclinical model so that rational strategies of treatment and prevention can be devised.

## Methods

### Animals

All mice in this study were offspring from a Del(16Dgcr2-Hira)1Rak (*LgDel*) colony maintained at The George Washington University. Wild-type (WT) and *LgDel* littermates were obtained by crossbreeding heterozygous C57BL/6N *LgDel* males with adult C57BL/6N WT females. Following genotyping by PCR (12) at weaning, 32 colony offspring were allocated to this study: 16 *LgDel* (11 males and five females) and 16 WT (10 males and six females). Mice were subsequently ear punched for identification and group housed (based on sex and litter) without experimental testing until approximately 3 months of age. At that time, 22 mice (11 *LgDel*: six males and five females; 11 WT: five males and six females) were shipped to the University of Missouri and following a 2-week quarantine period, were processed for fluoroscopic (13–15) and endoscopic (16–19) assessments of deglutition-related structure and function. The remaining 10 mice (5 *LgDel* and 5 WT, all males) were retained at The George Washington University for video surveillance of feeding and grooming activity using automated behavioral analysis (HomeCageScan 3.0; CleverSys Inc., Reston, VA) and Capture Star software (Version 1; CleverSys Inc.). Mice were housed in accordance with NIH and Institutional Animal Care and Use Committee guidelines, under standard local light/dark cycle conditions at The George Washington University (14/10 h) and the University of Missouri (12/12 h).

### Experimental Procedures

Mice underwent experimental procedures described below between 3 and 4 months of age, followed by euthanasia for post-mortem assessment of lung tissue and cranial bones. The genotypes of all mice were blinded until all data collection was completed; *unblinding occurred* following data entry for statistical analysis.

#### Fluoroscopic Assessment of Feeding and Swallowing

**Figure 1:**
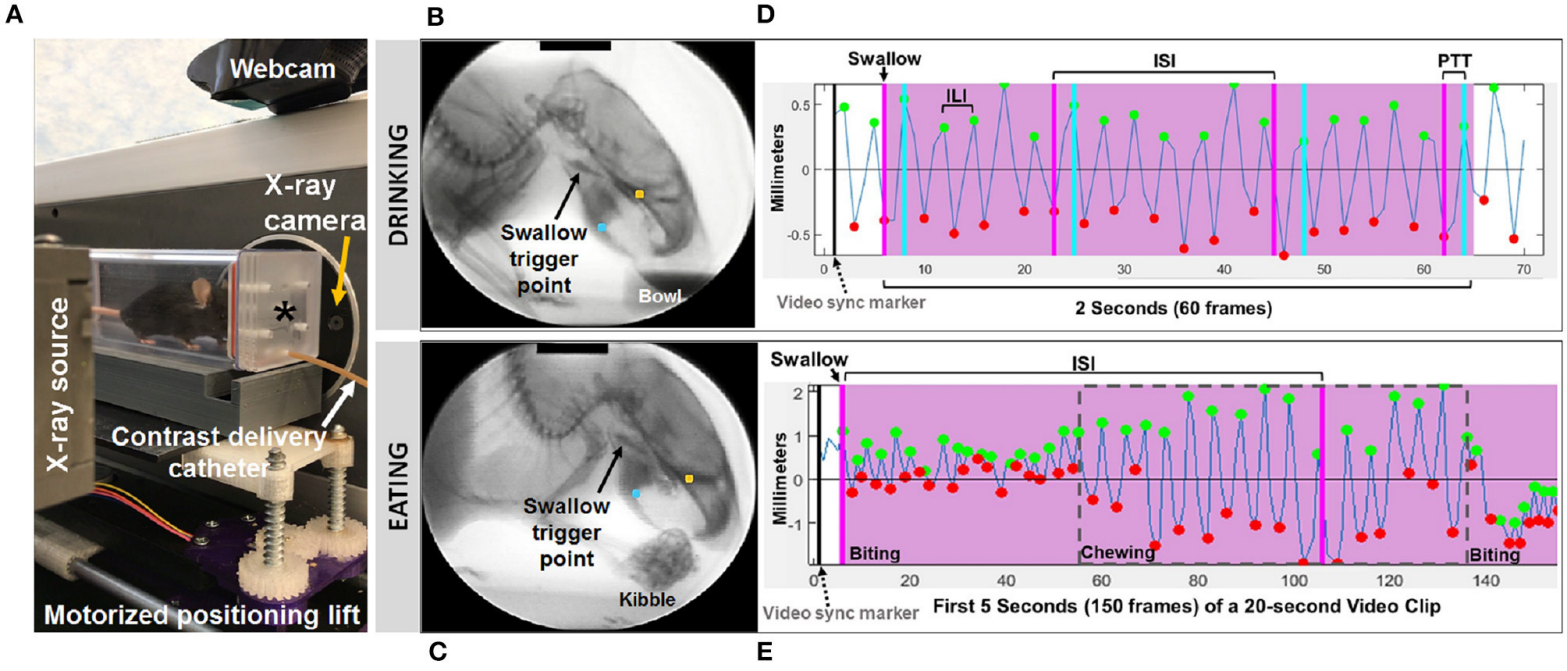
Fluoroscopic assessment of feeding and swallowing. **(A)** A mouse confined within a test chamber (asterisk) positioned in lateral view within our miniature fluoroscope. Representative radiographic images of a mouse voluntarily drinking liquid contrast from a bowl **(B)** and eating barium extruded kibble held in the forepaws **(C)**, with jaw tracking markers positioned on the upper (yellow) and lower (blue) jaw using our JawTrack™ software. Representative plots showing automated tracking of jaw open/close motion during drinking **(D)** and eating **(E)**, with labeled events of interest. Green and red dots indicate when the jaw is maximally opened vs. closed, respectively. Pink shaded box indicates region of interest for analysis (2 s for drinking, 20 s for eating), starting with a swallow event (pink line). Gray dashed box distinguishes rotary chewing from incisive biting patterns during eating. ILI, inter-lick interval; ISI, inter-swallow interval; PTT, pharyngeal transit time. Radiographic calibration marker (black line) = 10 mm.

Mice (*n* = 11 WT, 11 *LgDel*, mixed sexes, 3–4 months of age) underwent videofluoroscopic swallow study testing (VFSS) at the University of Missouri using custom equipment and an established protocol (13–15). Following 2-week behavioral conditioning to optimize performance, VFSS testing was performed separately for drinking vs. eating, spaced 1 week apart. For each test (drinking vs. eating), mice were individually subjected to ~2 min of low dose radiation (~30 kV and ~0.2 mA) using our miniaturized fluoroscope (The LabScope, Glenbrook Technologies, Randolph, NJ). The night prior to testing, mice were weighed (grams) and then underwent either a water restriction (12 h) to motivate voluntary drinking or a food restriction (4–6 h) to motivate voluntary eating, both in the home cage. A VFSS test chamber with one endcap removed was placed in the home cage overnight for mice to voluntarily explore; this same test chamber was used during VFSS testing the following morning. During testing, mice were enclosed within the test chamber and positioned within the lateral plane of the fluoroscope (Figure 1). For drinking, our established thin liquid oral contrast agent (Omnipaque, GE Healthcare, 350 mg iodine/mL; diluted to a 25% solution with deionized water and 3% chocolate flavoring) was administered via a custom syringe delivery device into a custom bowl, secured to the test chamber end-cap closest to the radiation source. For eating, peanut butter flavored kibble (circular shape, ~10 mm diameter ×5 mm thick) extruded with barium (40% weight/volume, manufactured in collaboration with AFB International, St. Charles, MO), which retains a dry, crunchy consistency, was used for fluoroscopic assessment of mastication-related behaviors. For each mouse, a half piece of kibble (~10 × 5 × 5 mm) was placed in the chamber bowl. Throughout testing, the fluoroscope was activated via foot pedal only when the mouse was actively drinking or eating, visualized in real-time using a webcam (Logitech, HD Pro C920) positioned above the test chamber. A custom, remote-controlled platform was used to maintain the mouse's head and neck in the fluoroscopy field of view while drinking and eating.

Approximately 30 s to 1 min of video was captured separately for drinking and eating episodes, digitally recorded at 30 frames per second (fps) and saved as AVI files. From these videos, five 2 s episodes of uninterrupted drinking and one 20 s episode of uninterrupted eating were identified; the start frame coincided with a swallow event, identified as abrupt movement of the bolus from the vallecular space (i.e., the stereotypical swallow trigger point in mice) to the esophagus. These “episodes” were spliced from the raw video using Pinnacle Studio (version 14; Pinnacle Systems, Inc., Mountain View, CA), with five frames added to each end to provide contextual information as needed during subsequent frame-by-frame analysis using our custom VFSS analysis software, JawTrack™. This software (© Copyright 2019 by The Curators of the University of Missouri) provides an interactive interface that permits automated tracking of jaw motion during drinking and eating in rodents based on the location of manually placed markers on the upper and lower jaw in the first frame of each video clip. The distance (in pixels) between the two markers is automatically converted into mm for each video frame, based on manual tracing of the 10 mm calibration marker at the top of the first video frame image displayed in the interface. Following jaw tracking, the interface displays a graph of cyclic jaw opening and closing motion (distance over time), synchronized with the video. Jaw tracking events (i.e., maximally opened or closed jaw) are manually reviewed and easily edited within the interface. Further, bolus flow events of interest (e.g., swallowing) can be manually added via makers within the jaw tracking graph. Once all events are edited/added, a set of VFSS metrics (Table 1) is automatically calculated and displayed in the interface as well as automatically exported into an Excel spreadsheet for subsequent use in statistical analysis. The only exception is mastication rate, which required manual identification of rotary chewing behaviors in the graphic display. Also of note, pharyngeal transit time was not included during eating, as bolus flow through the distal pharynx and proximal esophagus was typically obscured by the shoulders and arms while mice ate kibble from the forepaws.

**Table 1:** VFSS metrics and operational definitions.

**VFSS metrics**		**Operational definitions**	**Units**
Drinking	Lick rate	Number of jaw open/close cycles per second, calculated separately for each second of a 2 s video clip, then averaged.	#/s
	Inter-lick interval	Time between successive lick cycles throughout a 2 s video clip.	ms
	Swallow rate	Number of swallows in each second of a 2 s video clip, converted to a rate (swallows/second), then averaged.	#/s
	Inter-swallow interval	Time between successive swallow pairs throughout a 2 s video clip, then averaged.	ms
	Lick-swallow ratio	Number of jaw open/close cycles between each successive swallow pair throughout a 2 s video clip, then averaged.	n/a
	Pharyngeal transit time	Bolus flow time through the pharynx for each successive swallow, then averaged. The start frame is the “rest frame” that immediately precedes visible transfer of the bolus from the vallecula (swallow trigger point). The end frame is when the tail of the bolus enters the esophagus.	ms
	Jaw closing velocity	Speed at which the jaw closes during each jaw cycle throughout a 2 s video clip, then averaged.	mm/s
	Jaw opening velocity	Speed at which the jaw opens during each jaw cycle throughout a 2 s video clip, then averaged.	mm/s
Eating	Mastication rate	Number of jaw open/close cycles per second during three separate 1 s episodes of rotary mastication, then averaged.	#/s
	Swallow rate	Number of swallows in each second of a 20 s video clip, converted to a rate (swallows/second), then averaged.	#/s
	Inter-swallow interval	Time between successive swallow pairs throughout a 20 s video clip, then averaged.	s

*All video clips depict uninterrupted drinking or eating behaviors, beginning with a swallow event (i.e., rest frame immediately preceding bolus flow from the vallecula). For each mouse, five 2 s episodes of drinking and one 20 s episode of eating were analyzed via JawTrack™*.

#### Endoscopic Assessment of Upper Airway Structure and Function

Within 1 week after completing VFSS testing, the same 22 mice underwent transoral endoscopy for gross assessment of craniofacial structure and function using our established protocol and custom equipment (16–19). The night prior to endoscopy, mice were food restricted for 4–6 h to prevent post-prandial retention of food in the pharynx that may interfere with testing. Mice were anesthetized with ketamine-xylazine (90 mg/kg ketamine,11.25 mg/kg xylazine, subcutaneous injection) followed by a single dose of ketamine (1/2 the original dose) to maintain light sedation (i.e., only local limb movement in response to toe pinch) while secured in ear bars in dorsal recumbency within our custom murine endoscopy suite. Core body temperature was maintained at 37 ± 0.2°C using a rectal thermocouple (DC Temperature Control System; FHC, Bowdoin, ME). Mice spontaneously breathed room air during the entire procedure, which lasted ~30 min.

Endoscopy was performed using a miniature endoscope (sialendoscope; R11573A; Karl Storz). A custom laryngoscope was used to secure the endoscope to a custom micromanipulator, which permitted precise manual control. The tongue was gently retracted as the endoscope was guided via micromanipulator into the oral cavity, then slowly advanced to visualize the pharynx and larynx. The larynx was maintained in the endoscope field of view for approximately 10 s to visualize spontaneous abduction and adduction motion during each inspiratory and expiratory phase of the respiratory cycle, respectively. Using our previously published methods (19), we then assessed the laryngeal adductor reflex (LAR) by delivering up to five air puffs per mouse, targeting the arytenoid mucosa near the dorsal commissure. Air pulses (4 mm Hg, 250 ms duration) were delivered via the sialendoscope working channel using our custom air pulse generating device, with stimuli spaced at least 10 s apart. Responses were scored as present or absent. A present response was identified by abrupt, brief glottic closure (i.e., bilateral arytenoid medialization) immediately following air pulse delivery. The entire endoscopic procedure was video recorded at 30 fps and saved as MPEG files.

**Figure 2:**
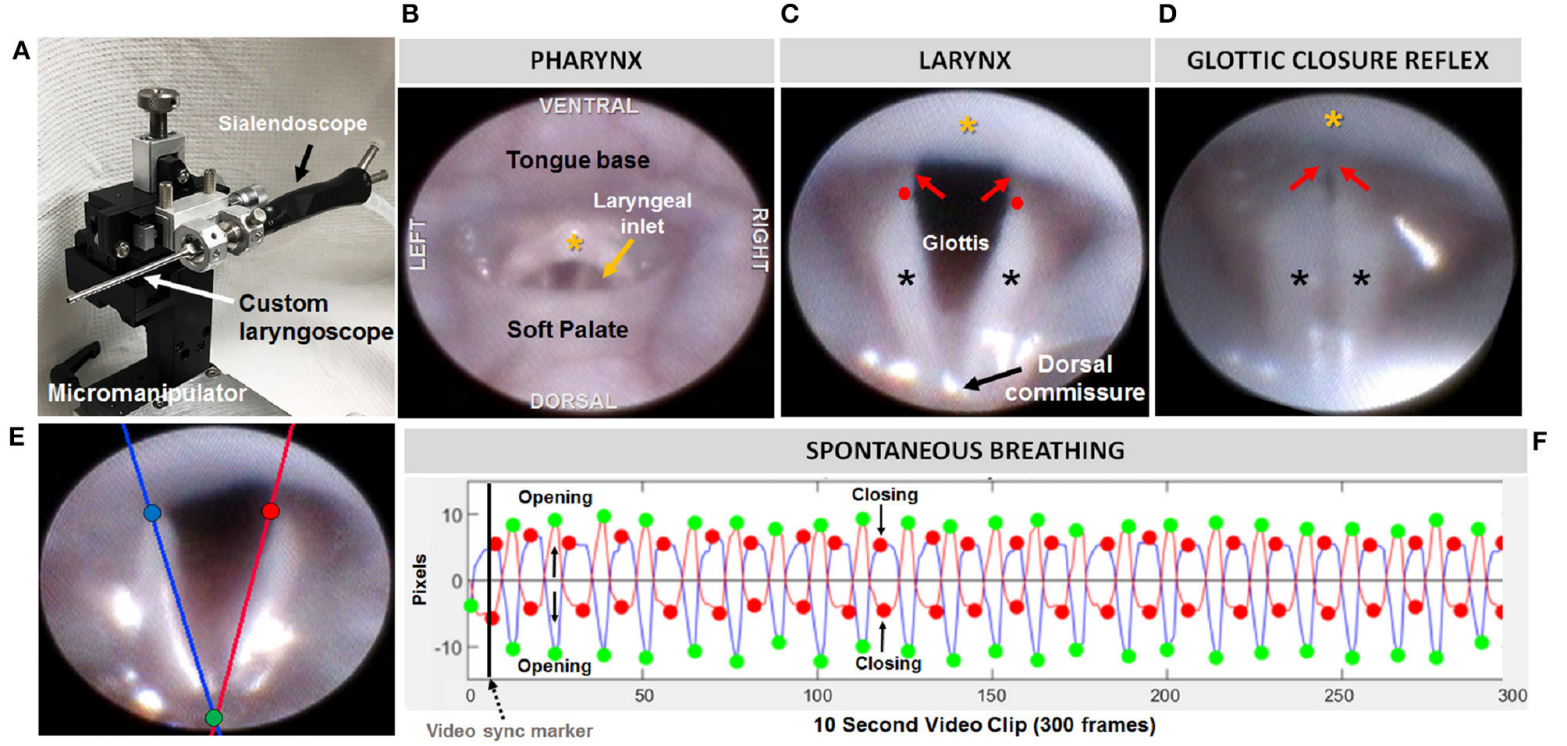
Endoscopic assessment of upper airway structure and function. **(A)** Modified sialendoscope with micromanipulator control for precise transoral insertion. Representative endoscopic images of a mouse pharynx **(B)** and larynx **(C)** with labeled structures. In mice, the glottal edge is formed predominantly by the arytenoids (black asterisks); the proportionately smaller vocal folds (red arrows) are nearly obscured by the epiglottis (yellow asterisk); red dots indicate the location of the arytenoid vocal process. **(D)** Air pulse stimulation of the arytenoid mucosa near the dorsal commissure evokes the glottic closure reflex (i.e., laryngeal adductor reflex, LAR), identified by brief, bilateral medialization of the arytenoids and vocal folds. **(E)** Tracking lines positioned along the left (blue) and right (red) glottal edge using our VFTrack™ software, based on the location of three manually placed markers within the software interface: blue (left arytenoid vocal process), red (right arytenoid vocal process), and green (dorsal commissure). **(F)** Representative plot showing automated tracking of glottal edge open/close motion during spontaneous breathing under light sedation. Green and red dots indicate when the glottis is maximally opened during inspiration vs. maximally closed during expiration, respectively.

At a later time, the videos were viewed via Pinnacle Studio (version 14; Pinnacle Systems) to identify gross structural and functional anomalies. LAR events were analyzed frame-by-frame to identify the start and end frame, which was used to calculate LAR duration (ms). From each video, a 10 s episode of uninterrupted vocal fold motion during spontaneous breathing was spliced from the raw video for objective analysis using our custom laryngeal motion analysis software, VFtrack™. This software (© Copyright 2017 by The Curators of the University of Missouri) provides an interactive interface that permits automated tracking of laryngeal motion during breathing, based on manually placed markers on the left and right glottal edge (near the vocal process) and dorsal commissure (midline between the arytenoids) in the first frame of each video clip. Using these three points, two separate lines are automatically drawn along the left and right glottal edge. The location of the left and right points is automatically adjusted to be equidistant from the dorsal commissure point, using the furthest left/right point as the reference. The adjusted points are then automatically tracked in all subsequent video frames and graphically displayed in the interface as a cyclic waveform representing the oscillatory motion of the larynx during breathing. Using the interface, glottal tracking events can be manually reviewed in synchrony with the video and edited as needed. Following manual review and editing, a set of laryngeal motion metrics (Table 2) is automatically calculated and displayed in the interface, as well as automatically exported into an Excel spreadsheet for subsequent use in statistical analysis. A summary of the entire endoscopic test and analysis process is shown in Figure 2.

**Table 2:** Laryngeal motion metrics and operational definitions.

**Laryngeal motion metrics**	**Operational definitions**	**Units**
Mean motion range ratio (MMRR)	Ratio of the right and left VF motion range (i.e., amplitude) during each respiratory cycle throughout a 10 s video clip, then averaged.	n/a
Open close cycle ratio (OCCR)	Ratio of the number of right and left VF motion cycles (i.e., frequency) throughout a 10 s video clip, then averaged.	n/a
Motion correlation coefficient (Mcorr)	Comparison of left and right VF motion direction (i.e., motion correlation coefficient) in each video frame throughout a 10 s clip, then averaged. Values range from −1 to 1, where values close to −1 represent a negative correlation (i.e., VF motion in opposite directions; normal function), values close to 1 represent a positive correlation (i.e., VF motion in the same direction; paradoxical motion), and values close to 0 represent minimal correlation (i.e., little to no VF motion).	n/a
VF angle	Measurement of VF maximum and minimum angle for each respiratory cycle throughout a 10 s video clip, then averaged.	degrees
Respiratory rate	Number of VF motion cycles per minute throughout a 10 s video clip, then averaged. VF abduction = inspiration; VF adduction = expiration.	#/min

*For each mouse, one 10 s video clip of spontaneous breathing under anesthesia was analyzed via VFtrack™*.

#### Craniofacial Imaging

While still anesthetized from endoscopy, photographic (Apple iPhone 6 Plus) and radiographic (LabScope) images were obtained for gross assessment of craniofacial structures and features. Mice were photographed from the front, left lateral, and right lateral positions, followed by fluoroscopic imaging in the lateral and axial planes. Images of *LgDel* mice were compared side-by-side with WT mice to identify visibly obvious abnormalities in craniofacial structure and symmetry.

#### Post-mortem Assessment of Lung Tissue and Cranial Bones

Following imaging and while still anesthetized, mice were euthanized by pentobarbital overdose (390 mg/ml + sodium phenytoin 50 mg/ml, intraperitoneal injection), followed by cardiac perfusion with saline and then 4% paraformaldehyde (PFA). The lungs (with trachea attached) and skulls were collected and shipped to The George Washington University on dry ice for processing. Lungs were washed in phosphate-buffered saline, equilibrated in 30% sucrose, and then embedded in optimal cutting temperature (OCT) compound. Frozen lung tissue was sectioned at 20 microns via cryostat (Leica CM1950) and stained with Hematoxylin and Eosin (H&E). Images were acquired using an Olympus BX63 Upright Microscope equipped with a DP80 digital camera and cellSens imaging software using the 10X and 20X objectives. Hemorrhages were digitally quantified in Adobe Photoshop (20). Five images were taken for each sample, each adjusted in Adobe Photoshop using the following methods: (1) the Magic Wand tool was used to select and remove the background from the image; (2) under the Hue/Saturation tool, the red channel was selected and increased to +100; (3) the blue channel was selected and maximally decreased, and the lightness was increased to +100; and (4) the brightness and contrast were changed to 150 and 100, respectively. These color adjustments isolated the darker red/purple hues of blood vessels and clumps of neutrophils. The threshold was then set to 130 to completely isolate the inflamed pixels. An inflammation ratio for each image was calculated by comparing the number of pixels within the threshold and the total number of pixels before editing. The five inflammation ratios per sample were averaged together to obtain a representative inflammation ratio for each mouse.

For bone analysis, fixed cranial bones were isolated by multiple digestions (3–4 days each, until tissue was removed, over a period of ~3 weeks) with proteinase K (200 μg/ml) at 60°C in buffer (20 mM Tris, 10 mM CaCl2, 400 mM NaCl, 1% Sodium dodecyl sulfate, pH 8.0). Bones were imaged on a Leica M420 microscope with a 5MP digital camera. Mandibles were imaged laterally, and pixel measurements between cardinal points were made in Adobe Photoshop and converted to millimeter measurements by scaling to a micrometer imaged in the same imaging session.

#### Video Surveillance of Feeding and Grooming Activity

**Figure 3:**
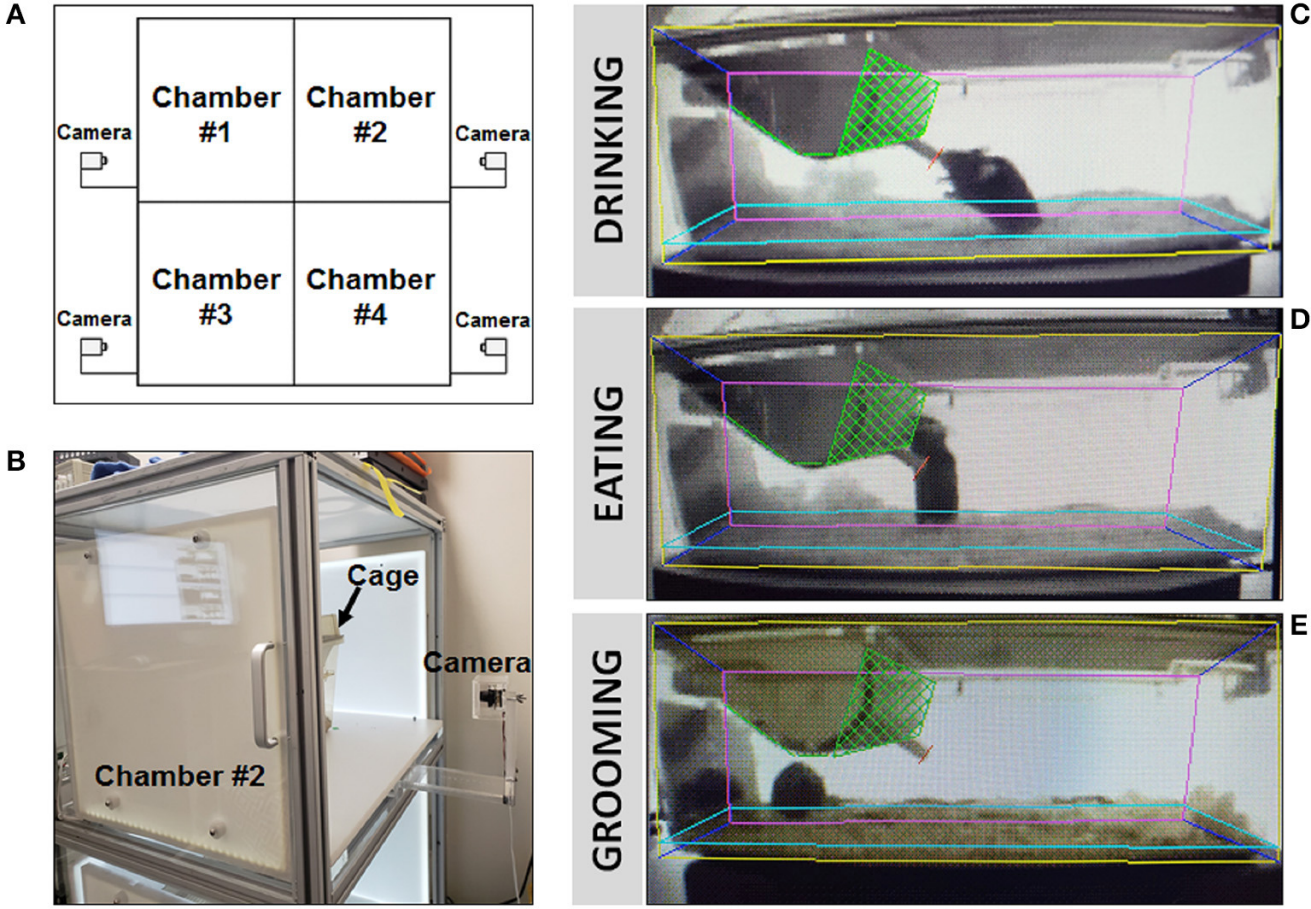
Behavioral video surveillance of murine activity via HomeCageScan. **(A)** Schematic of CleverSys Inc. HomeCageScan chamber and camera set-up made up of four separate chambers, each with its own camera for behavioral recording. One mouse/cage was placed into each chamber, up to 4 animals at a time to be analyzed. **(B)** Enlarged image for detail of the cameras used to record all behaviors attached to each chamber. **(C–E)** Specific parameters for each standard size cage were manually defined prior to recording in order to ensure accurate analysis: yellow—area of the proximal lateral cage wall, pink—area of the distal lateral cage wall, dark blue—area of the front and back cage walls, light blue—area and height of the bedding, green—food container, red—drinking spout. All three images were taken from the same animal. **(C)** Still image of “drinking” behavior recorded and analyzed by the software. **(D)** Still image of “eating” behavior recorded and analyzed by the software. **(E)** Still image of “grooming” behavior recorded and analyzed by the software.

A separate cohort consisting of 10 male mice (5 WT, 5 *LgDel*, 3–4 months of age) collected from multiple litters, was assessed using an automated behavioral analysis system (HomeCageScan 3.0; CleverSys Inc., Reston, VA) and Capture Star software (Version 1; CleverSys Inc.) that permits real-time detection and analysis of a variety of unconstrained rodent behaviors (21). For this study, we focused on detection and analysis of drinking, eating, and grooming behaviors for comparison with non-oromotor-based behaviors (Figure 3). Testing entailed placing individual mice into a clean shoebox-style acrylic cage with a filter top. Within each cage, a wire top feeder provided free access to standard rodent pellets in a U-shaped hopper and water from a standard spout bottle. Each cage was placed into one of the four chambers (stacked 2 X 2) within the monitoring system, each equipped with one infrared camera positioned exterior to the right or left side of the cage, depending upon chamber assignment, for side-view recording. The position of all four cameras was adjusted within each chamber to maintain a consistent field of view within and between cages. To maximize visibility within the cage, enrichment material was limited to a thin layer of cobb bedding on the cage floor and half of a nestlet (i.e., nesting material). Mice were acclimated to the cage for 24 h, followed by 72 consecutive hours of video recording (30 fps, MPG file format) and real-time detection and analysis of drinking, eating, and grooming activity (frequency and duration; Table 3). Prior to recording, the following parameters were manually defined within the software: location of the food hopper and waterspout, and interior cage perimeter (i.e., free-space accessible to the mouse). It should be noted that all mice appeared healthy prior to and following the recording, with no evidence of barbering, hair loss, or skin lesions due to chewing. Additionally, eating and drinking occurred while the mice were rearing on hind legs due to the location of the food hopper and waterspout, as shown in Figure 3, which allowed the software to readily detect these behaviors for analysis. For each mouse, the automatically detected and analyzed drinking, eating, and grooming data (frequency and duration) from the 72 h of video recording were exported to Excel as three 24 h periods (bins), each including chronological event classification (drinking, eating, or grooming), along with the corresponding timestamp, frequency, and duration of each event. At a later time, the data were “spot checked” for accuracy at ~6 h intervals by manually comparing the automated event classification with the corresponding timestamp in the video recording. Data from the three 24 h bins were averaged for each mouse for statistical analysis.

**Table 3:** HomeCageScan metrics and operational definitions.

**HomeCageScan metrics**			**Operational definitions**	**Units**
Oromotor	Drinking	Frequency	Number of times that the animal's snout is in close proximity to the defined water spout in the act of drinking.	events/bin
		Duration	The length of time the animal spends drinking as analyzed by the animal's snout being within close proximity to the defined water spout starting from a non-drinking position, to active drinking, until finished.	seconds/bin
	Eating	Frequency	Number of times that the animal's snout is at/near the defined food hopper in the act of eating.	events/bin
		Duration	The length of time the animal spends eating as analyzed by the animal's snout being at/near the defined food hopper from a non-eating position, to active eating, until finished.	seconds/bin
	Grooming	Frequency	The number of times the animal's snout is in close proximity to its body and the body deforms into a grooming position while making specific grooming movements with its head, paws, and body.	events/bin
		Duration	The length of time an animal spends in a grooming position, with the animal's snout in close proximity to its body and making specific grooming movements with its head, paws, and body.	seconds/bin
Non-Oromotor	Walking Slowly	Frequency	The number of times that the animal makes any sideways movement that does not have a definite directional component.	events/bin
	Come Down	Duration	The length of time an animal spends moving from a fully reared up position to a low position.	seconds/bin
	Hang vertically from hang cuddled	Duration	The length of time and animal spends moving from a hang cuddled position to a hang vertical position (note: the hang cuddled position involves the animal having all four limbs at the top of the cage in a horizontal position).	seconds/bin

*Each “bin” represents a 24 h period of data collection*.

#### Statistical Analysis

After verifying a normal data distribution for each variable, independent samples *t*-tests were used to explore differences between the two genotypes (WT and *LgDel*), using averaged data when applicable. Outliers were identified but not removed from the dataset. Statistical analyses were performed using IBM SPSS Statistics 24. Variability within genotype was reported as the mean ± standard error of mean (SEM) for each variable, and two-sided *p* values of 0.05 or less were considered statistically significant. Mandibular measurements were assessed by 2-way ANOVA (genotype x side, GraphPad PRISM) to account for measurements of both left and right bones.

## Results

### Fluoroscopic Assessment of Feeding and Swallowing

**Figure 4:**
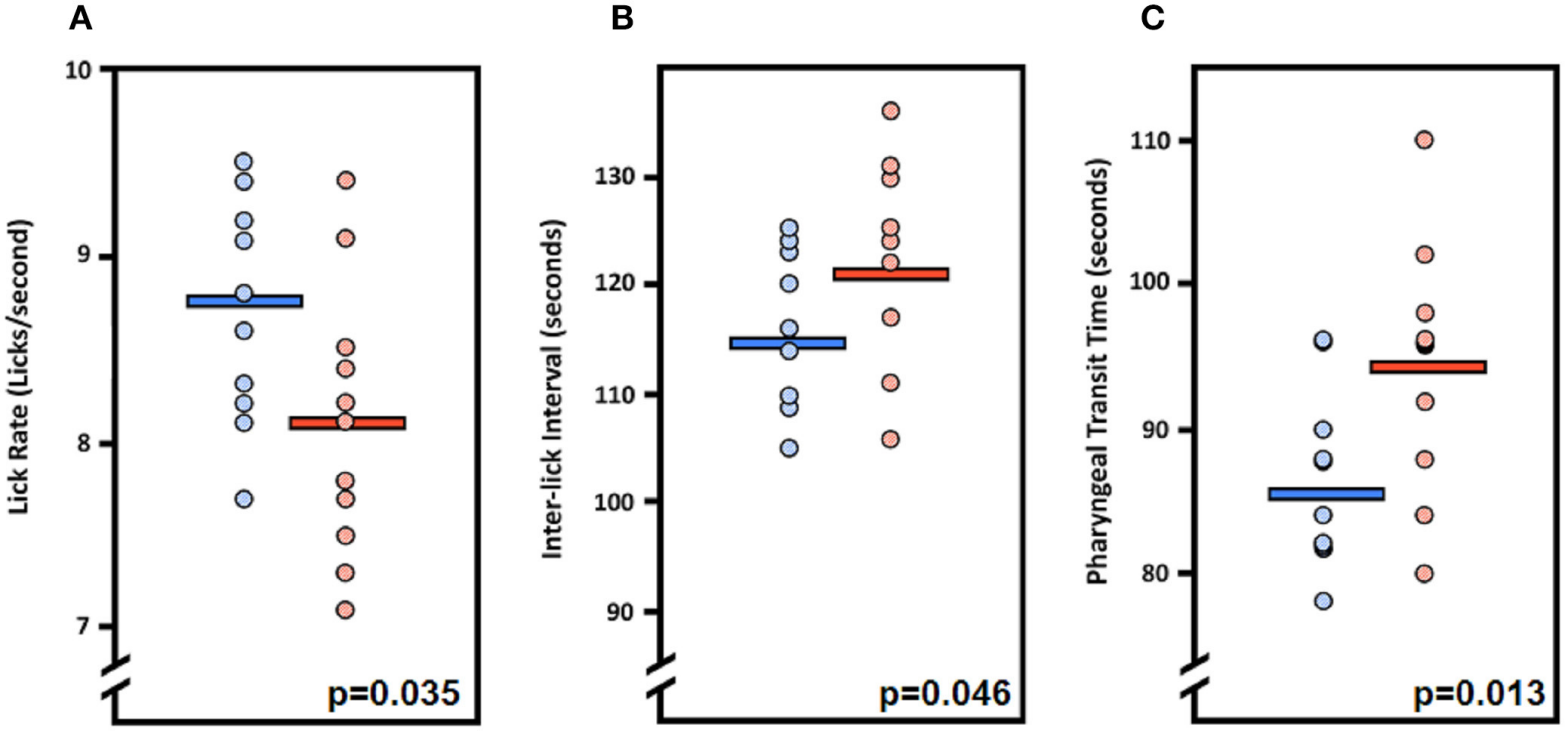
Fluoroscopic evidence of feeding and swallowing deficits in *LgDel* mice. Analysis of fluoroscopic videos using our JawTrack™ software revealed that three of the eight VFSS metrics investigated were statistically significant between *LgDel* mice (red) and WT controls (blue). Specifically, *LgDel* mice had **(A)** slower lick rates, **(B)** longer inter-lick intervals, and **(C)** longer pharyngeal transit times when voluntarily drinking thin liquid contrast.

We first asked whether the oral or pharyngeal phases of feeding and swallowing in *LgDel* mice differed from their WT counterparts. All 22 mice subjected to VFSS testing voluntarily participated, resulting in 110 drinking-based video clips (2 s each) and 22 eating-based video clips (20 s each) for frame-by-frame analysis of VFSS metrics (Table 1) using JawTrack™. Body weight prior to VFSS testing was not significantly different between groups (*p* = 0.373; WT: 22.39 ± 0.55; *LgDel*: 21.83 ± 0.26). We analyzed both male and female mice; however, we have not separated the samples by sex for this study. Some behavioral sex differences have been described in individuals with 22q11DS: males tend to be more withdrawn, have more somatic complaints, and are more likely to have anxiety and depression than females (22). Nevertheless, the incidence of dysphagia and other airway abnormalities in 22q11DS does not differ between males and females (1, 10, 23–26). In addition, our previous research with adult C57BL/6J mice revealed no significant differences in swallowing function between sexes (13). Compared to WT mice, *LgDel* mice had altered swallowing behaviors during drinking (Table 4; Figure 4). Specifically, *LgDel* mice had significantly slower lick rates (*p* = 0.035; WT: 8.71 ± 0.18; *LgDel*: 8.07 ± 0.22; Figure 4A), longer inter-lick intervals (*p* = 0.046; WT: 114.43 ± 2.30; *LgDel*: 121.91 ± 2.65; Figure 4B), and longer pharyngeal transit times (*p* = 0.013; WT: 85.82 ± 1.91; *LgDel*: 94.36 ± 2.50; Figure 4C). All other drinking- and eating-based VFSS metrics were not statistically different between genotypes (*p* > 0.05; Table 4). Thus, there are significant differences in distinct, measurable aspects of the oral and pharyngeal phases of feeding and swallowing in adult *LgDel* mice.

**Table 4:** VFSS summary statistics.

**VFSS metrics**		***p*-value**	**Mean (±SEM)**	
			**WT**	***LgDel***
Drinking	Lick rate (#/s)	**0.035**	8.71 (0.18)	8.07 (0.22)
	Inter-lick interval (ms)	**0.046**	114.43 (2.30)	121.91 (2.65)
	Swallow rate (#/s)	0.508	1.68 (0.11)	1.77 (0.08)
	Inter-swallow interval (ms)	0.356	752.91 (56.22)	690.18 (35.35)
	Lick-swallow ratio	0.228	4.47 (0.36)	3.85 (0.34)
	Pharyngeal transit time (ms)	**0.013**	85.82 (1.91)	94.36 (2.50)
	Jaw closing velocity (mm/s)	0.255	14.39 (0.77)	13.19 (0.67)
	Jaw opening velocity (mm/s)	0.883	13.71 (0.71)	13.55 (0.79)
Eating	Mastication rate (#/s)	0.852	8.26 (0.31)	8.167 (0.42)
	Swallow rate (#/s)	0.228	0.31 (0.08)	0.27 (0.02)
	Inter-swallow interval (s)	0.840	4.01 (0.60)	4.17 (0.43)

*Bold p-values denote statistical significance (< 0.05); SEM, standard error of the mean; VFSS, videofluoroscopic swallow study; WT, wild-type*.

### Transoral Endoscopy

**Figure 5:**
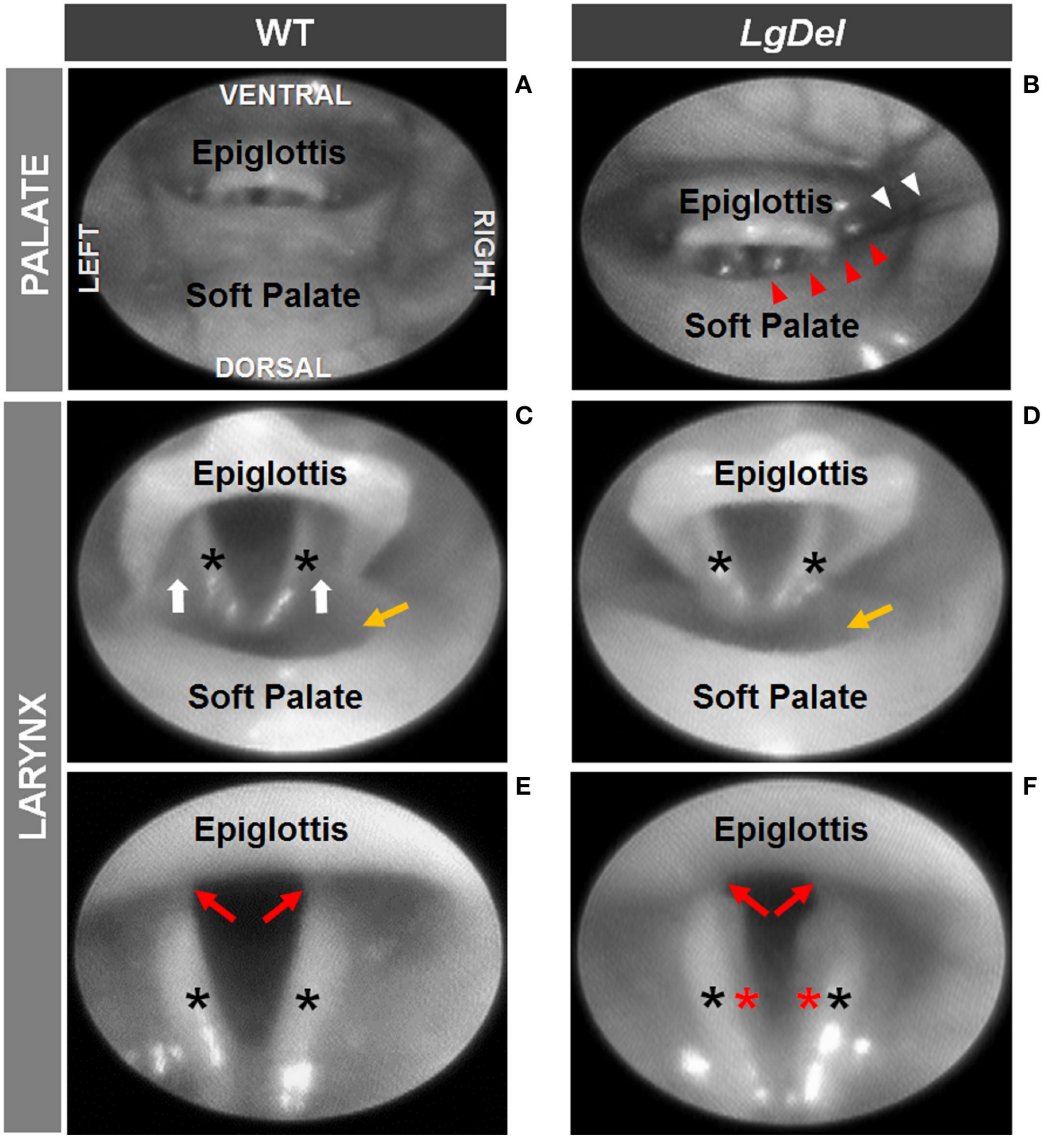
Endoscopic evidence of palatal and laryngeal anomalies in *LgDel* mice. Representative images showing advancement of the endoscope into the pharynx **(A,B)** and laryngeal inlet **(C,D)** to visualize the glottis **(E,F)**. Compared to WT mice **(A,C,E)**, *LgDel* mice displayed several minor structural anomalies, including soft palate asymmetry (red arrowheads), and in this mouse, strands of fur (white arrowheads) were found lodged within the laryngeal inlet **(B)**; narrowed epiglottis with visibly absent aryepiglottic folds **(D)**; and extraneous mucosa (red asterisk) along the medial edge of the arytenoids **(F)**. Black asterisks, arytenoid mucosa; white arrows, aryepiglottic folds; red arrows, vocal folds; yellow arrow, laryngeal pouch. Images were adjusted for color, brightness, and contrast to enhance visualization of key features.

We next asked whether oropharyngeal dysmorphology accompanies these functional differences in *LgDel* adult feeding and swallowing. Minor structural anomalies of the palate and larynx were identified in four of the 11 *LgDel* mice (36%) that underwent transoral endoscopic assessment. All of the WT mice appeared structurally normal. Specifically, one *LgDel* mouse had an asymmetric soft palate, two had extraneous laryngeal mucosa along the medial edge of the glottis, and another had a narrowed larynx without any visible aryepiglottic folds (Figure 5). Laryngeal adductor reflex (LAR) testing was successful in only eight mice (4 WT and 4 *LgDel*), mainly attributed to the laryngoscope diameter (2.0 mm outer diameter) being slightly too large to pass through the laryngeal inlet for targeted air pulse delivery to the dorsal commissure of the larynx. The LAR was evoked in all 4 WT mice but only three of the four *LgDel* mice. For the seven mice with LAR responses, no difference in LAR duration was identified between WT and *LgDel* mice (*p* = 0.197). VFtrack™ analysis of the 10 s endoscopic video clips revealed that laryngeal motion metrics were not significantly different between WT and *LgDel* mice (*p* > 0.05), as summarized in Table 5. In other words, laryngeal motion in *LgDel* mice was bilaterally symmetric during spontaneous breathing under light anesthesia, without detectable aberrations in motion range or frequency. Thus, structural anomalies of the palate, glottis and larynx, at moderate penetrance, accompany functional disruption feeding and swallowing in *LgDel* mice. Nevertheless, the consequences of these anomalies for baseline laryngeal reflexes and function during breathing are uncertain; few differences were detected between *LgDel* and WT for these measures.

**Table 5:** Laryngeal motion summary statistics.

**Laryngeal motion metrics**	***p*-value**	**Mean (±SEM)**	
		**WT**	***LgDel***
Mean motion range ratio (MMRR)	0.194	0.90 (0.06)	0.77 (0.07)
Open close cycle ratio (OCCR)	0.236	0.94 (0.05)	1.01 (0.02)
Motion correlation coefficient (Mcorr)	0.952	−0.86 (0.23)	−0.85 (0.04)
Average VF angle (degrees)	0.253	33.57 (1.40)	30.60 (2.10)
Respiratory rate (#/min)	0.092	165.26 (6.48)	143.61 (10.40)

*Bold p-values denote statistical significance (< 0.05). WT, wild-type*.

### Craniofacial Imaging

**Figure 6:**
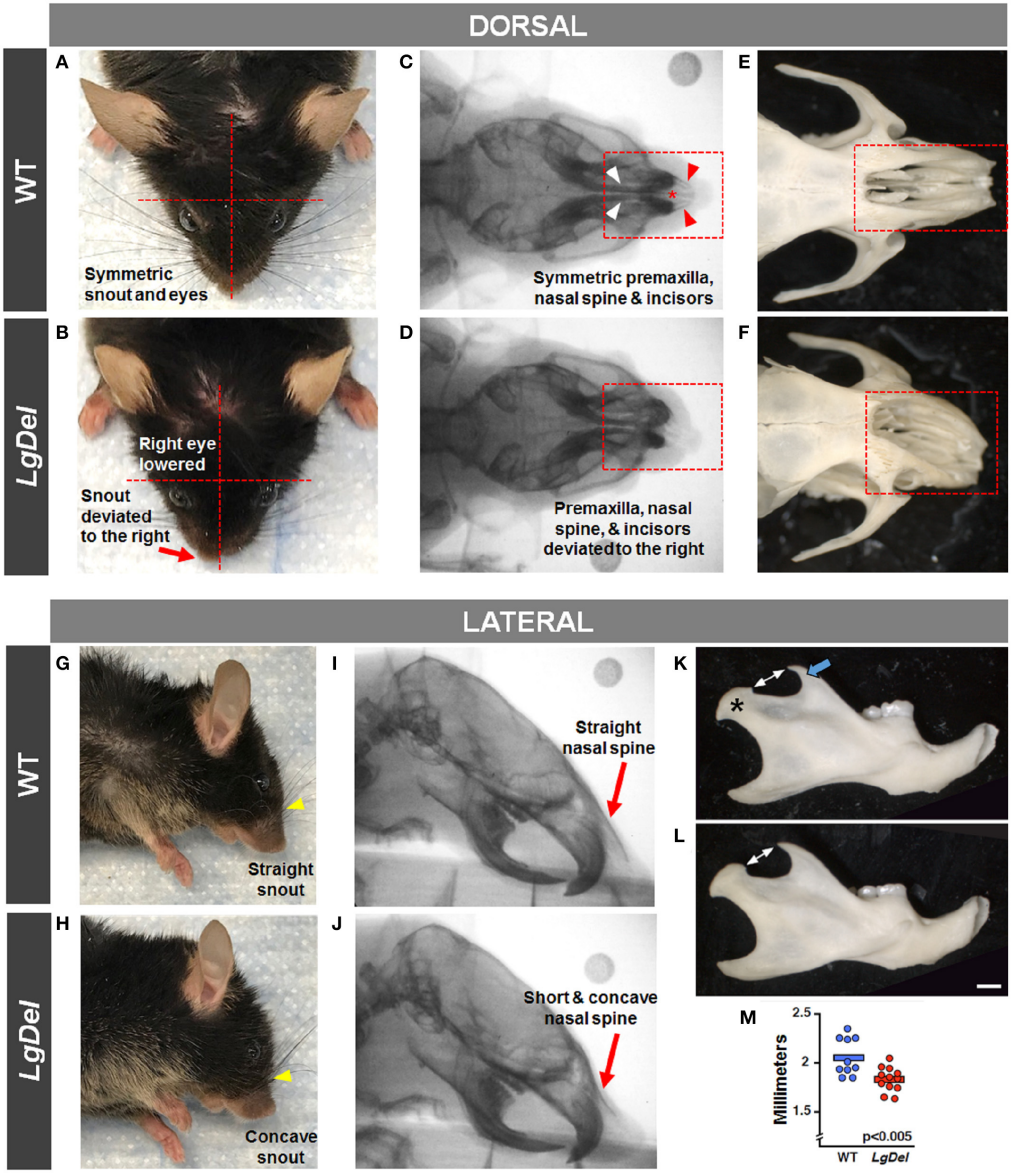
Craniofacial anomalies in *LgDel* mice identified via facial photographs, skull radiographs, and bone morphology. Representative images of a WT mouse in dorsal and lateral view **(A,G)** and skull bones **(C,E,I)** showing symmetric facial features. Some *LgDel* mice had facial asymmetry involving the eyes and snout **(B,H)**, and skull abnormalities involving the nasal spine, premaxilla, and incisors **(D,F,J)**. The mouse depicted here displayed all of these abnormalities; however, this phenotype had low penetrance. Representative examples of right mandible from WT **(K)** and *LgDel* **(L)** mice showing difference in morphology of the coronoid process (blue arrow) and condyle (asterisk). **(M)** Quantification shows *LgDel* mice have a significantly shorter distance between the coronoid process and the head of the mandible than their WT counterparts (*p* < 0.005). Scale bar = 1 mm.

It seemed possible that partially penetrant, but significant, oropharyngeal functional and structural anomalies in *LgDel* adult mice might occur in concert with extrinsic craniofacial anomalies. Facial photography and skull radiographs revealed structural anomalies of the eyes, premaxilla, nasal spine, incisors, and/or snout in two of the 11 *LgDel* mice (18%; Table 6; Figure 6). One of these mice was previously identified via endoscopy as having soft palate asymmetry. This brings the final count to five of the 11 *LgDel* mice (45%) identified with anomalies based upon assessment of craniofacial structure and function. To confirm these *in vivo* assessments we isolated the mandible, nasal, frontal and zygomatic bones of the dorsal skull of one of these mice, and saw significant bone dysmorphology that parallels the live craniofacial malformations (Figure 6). Thus, in agreement with initial measures of quantitative changes in the size and structure of the mandible in juvenile *LgDel* mice (7), there is evidence of variable extrinsic craniofacial dysmorphology in *LgDel* adults.

**Table 6:** Individual *LgDel* mice with craniofacial structural and functional anomalies.

**Mouse**	**Palate**	**Larynx**	**Face**	**LAR**
1	__	Extraneous laryngeal mucosa along the medial edge of the glottis	__	__
2	__	Extraneous laryngeal mucosa along the medial edge of the glottis	__	Absent
3	Soft palate asymmetry	__	Snout asymmetry	NT
4	__	__	Asymmetry of the eyes, premaxilla, nasal spine, incisors, and snout	NT
5	__	Narrowed larynx, aryepiglottic folds not visible	__	NT

*LAR, laryngeal adductor reflex (i.e., glottic closure reflex); NT, not tested*.

To assess whether the mandibles of the *LgDel* animals we analyzed were morphologically distinct from our WT sample for this study, we performed a multi-point morphometric assay, measuring the distance between cardinal points (7). The results of these measures that assess dorsal-ventral and anterior-posterior lengths in this relatively small sample of adult male mice of both genotypes were significantly more variable than in the much larger cohort of younger mice we analyzed previously (7), and did not reach statistical significance. On further inspection, there was one morphological distinction between *LgDel* and WT mandibles: the shape and size of the mandibular notch (i.e., the curved depression between the coronoid process and the head of the mandible) appeared altered. An additional measurement of the distance between the tip of the coronoid and the mandibular head confirmed this difference (*p* < 0.005 by two-way ANOVA; Figure 6).

### Video Surveillance of Feeding and Grooming Activity

**Figure 7:**
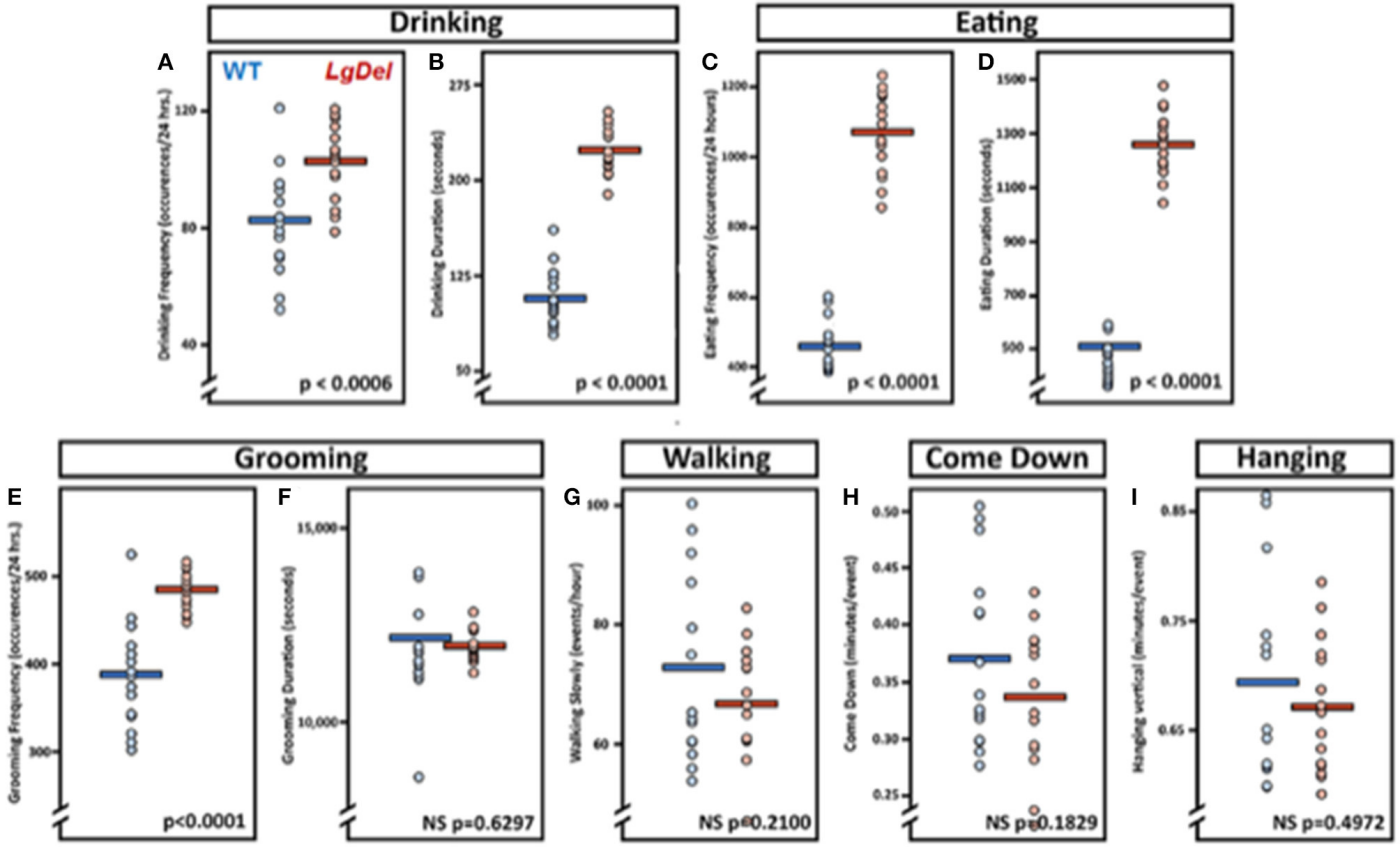
Feeding and grooming activity is altered in *LgDel* mice. Behavioral activity recorded via CleverSys Inc., HomeCageScan system. All animals were recorded for 72 consecutive hours and subsequently analyzed in 24-hour bins. Each bin was plotted for all animals along with SEM and mean values (WT, *N* = 5; LD, *N* = 5). Compared to WT mice, *LgDel* mice had a significantly higher drinking frequency **(A)**, longer drinking duration **(B)**, higher eating frequency **(C)**, and longer eating duration **(D)**. In addition, grooming frequency occurred at a significantly higher rate for *LgDel* mice compared to WT animals **(E)**; however, grooming duration was not significantly different between groups **(F)**. Non-oromotor behaviors, such as walking **(G)**, come down duration **(H)**, and duration of hanging vertically from a cuddled position **(I)**, did not differ between the *LgDel* and WT mice.

The evidence for selective disruption of feeding and swallowing mechanics, and related anatomical anomalies, suggested that ongoing feeding or other orofacial behaviors observed in mice housed in standard conditions without any manipulations or special modes of measurement might differ in *LgDel* vs. WT mice. We used an automated video recording and coding system (HomeCageScan; see Methods) to observe and quantify natural feeding-related behaviors with no additional intervention. We recorded several spontaneous behaviors in the home cage over a period of 72 h. Review of the quantitative data and corresponding video recording at ~6 h intervals for each mouse revealed that automated detection/classification of drinking, eating, and grooming behaviors via HomeCageScan was accurate. All three classes of behaviors were altered in *LgDel* compared to WT mice, based upon the reported mean and SEM values and corresponding statistical analysis (Table 7; Figure 7). The drinking frequency (events/bin) was 1.2 times higher for *LgDel* animals compared to WT mice (*p* = 0.0006; WT: 82.32 ± 4.8; *LgDel*: 102.72 ± 3.36; Figure 7A), with an associated 2-fold increase in drinking duration (seconds/24 h; *p* < 0.0001; WT: 107.40 ± 6.00; *LgDel*: 223.2 ± 4.80; Figure 7B). Similarly, the eating frequency was 2.3 times higher for *LgDel* animals compared to WT mice (*p* < 0.0001; WT: 456.00 ± 19.20; *LgDel*: 1,070.88 ± 30.72; Figure 7C), with an associated 2.8-fold increase in eating duration (*p* < 0.0001; WT: 442.20 ± 18.60; *LgDel*: 1,257.60 ± 30.60; Figure 7D). Disparities in grooming habits were also observed between the two groups of mice. While the grooming frequency was 1.2 times higher for *LgDel* animals compared to WT mice (*p* < 0.0001; WT: 386.64 ± 15.36; *LgDel*: 484.56 ± 5.52; Figure 7E), the grooming duration was not found to be significantly different between the two groups of animals (*p* = 0.6297; WT: 12,168.60 ± 427.80; *LgDel*: 11,948.40 ± 103.80; Figure 7F). We also evaluated non-oromotor behaviors including walking slowly, come down duration, and duration of hanging vertically from a cuddled position, which did not differ in the *LgDel* animals vs. WT mice (Figures 7G–I). It is apparent that the *LgDel* mice have ongoing challenges in drinking, eating, and grooming, all of which require oromotor coordination, in their standard environment.

**Table 7:** Feeding and grooming activity summary statistics.

**VFSS metrics**		***p*-value**	**Mean (±SEM)**	
			**WT**	***LgDel***
Drinking	Frequency (events/hour)	**0.001**	3.43 (0.20)	4.28 (0.14)
	Duration (minutes/24 h)	**<0.0001**	1.79 (0.10)	3.72 (0.08)
Eating	Frequency (events/hour)	**<0.0001**	19.00 (0.80)	44.63 (1.28)
	Duration (minutes/24 h)	**<0.0001**	7.37 (0.31)	20.96 (0.51)
Grooming	Frequency (events/hour)	**<0.0001**	16.11 (0.64)	20.19 (0.23)
	Duration (minutes/24 h)	0.630	202.81 (7.13)	199.14 (1.73)

*Bold p-values denote statistical significance (< 0.05); SEM, standard error of the mean; WT, wild-type*.

### Post-mortem Assessment of Lung Tissue and Cranial Bones

**Figure 8:**
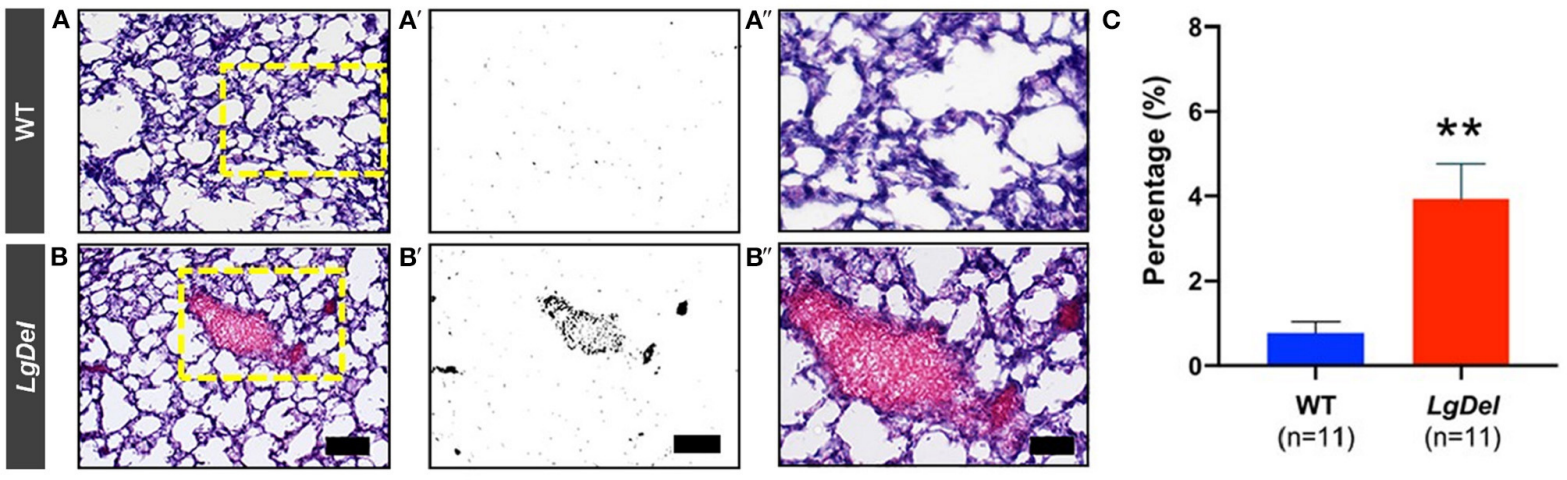
Inflammation of lung tissue in *LgDel* mice. Representative image of H&E stained lung tissue from a WT **(A)** and *LgDel* **(B)** mouse taken with the 10X objective; scale bar = 100 microns. The *LgDel* sample shows evidence of inflammation, including pools of erythrocytes (red staining). **(A',B')** Processed images showing isolation of pixels with pools of erythrocytes in *LgDel* but not WT samples. **(A”,B”)** Magnification of boxed regions in **(A,B)** showing greater detail of inflammation found in the *LgDel* lungs. Images were taken with the 20X objective; scale bar = 50 microns. **(C)** Graph showing significantly increased lung inflammation in *LgDel* compared to WT littermates. **p < 0.01.

Our previous studies established increased inflammation and the presence of milk proteins in the lungs as a signal of aspiration-based dysphagia in neonatal *LgDel* mice (6, 7). In H&E stained sections, lung inflammation appears as increased blood vessel dilation with pooling of blood in the tissue (27). To evaluate inflammation in the lungs of mice previously evaluated by fluoroscopic assessment of feeding and swallowing, dissected lungs were assessed for histological evidence of inflammation (Figure 8). *LgDel* lungs showed significantly greater evidence of inflammation, including dilated blood vessels and cellular accumulations of eosin stained proteins and erythrocytes. We found a >4-fold increased lung inflammation in *LgDel* compared to control mice (*p* = 0.0016; WT: 0.78 ± 0.13%; *LgDel*: 3.93 ± 0.85%; Figure 8). There was no correlation between lick rate and lung inflammation as determined by plotting lick rate vs. lung inflammation, followed by linear regression. For example, the most affected *LgDel* mutant for lick rate was the most normal of the mutants in terms of inflammation but the second highest *LgDel* mouse for lick rate. The *LgDel* mouse with the most apparent craniofacial abnormalities had a lick rate of 7.2 Hz and was near the mean in terms of inflammation (3.34%).

To assess the mandibles, we performed a multi-point morphometric assay to measure the distance between cardinal points, as performed previously (7), using the male cohort of tested mice. The results of these measures—designed to assess dorsal-ventral and rostral-caudal lengths—were significantly more variable than a previously tested younger cohort and did not reach statistical significance. On further inspection, it appeared that there was a morphological distinction between *LgDel* and WT mandibles, particularly in the shape of the sigmoid (i.e., mandibular) notch. An additional measurement of the distance between the tip of the coronoid process and the condyle revealed a significant difference between groups (*p* < 0.005). Images from isolated skull bones are included in Figure 6, which illustrate dysmorphology similar to our findings via facial photographs and skull radiographs.

## Discussion

We characterized functional, structural, and baseline behavioral correlates of feeding and swallowing in adult *LgDel* mice to determine if dysphagia recognized perinatally in *LgDel* pups is followed by sustained difficulties in feeding and swallowing in maturity. We found that lick rate is slower and the inter-lick interval is longer in *LgDel* adult mice, both of which are correlates of impaired feeding and oral stage dysphagia. The increase in pharyngeal transit time is an indicator of motility issues during the pharyngeal stage of swallowing. These functional changes are accompanied by variably penetrant oropharyngeal dysmorphology and extrinsic craniofacial anomalies in adult *LgDel* mice. These specific disruptions in feeding, swallowing, and related oropharyngeal and craniofacial structures were paralleled by altered homeostatic drinking, eating, and grooming, all of which require oromotor coordination. Finally, *LgDel* mice had far more frequent signs of lung inflammation consistent with food and/or liquid aspiration than WT counterparts. Together, these anomalies demonstrate that the developmental disruptions associated with perinatal feeding and swallowing difficulties in *LgDel* mouse pups are maintained, resulting in a high frequency of feeding and swallowing difficulties in adulthood.

### Persistent Feeding Difficulties and Oropharyngeal Dysphagia

Deficits in lick rate, rhythm (inter-lick interval), and pharyngeal transit time in *LgDel* mice are indicative of tongue dysfunction, which corresponds with our previous finding of altered CN XII neurodevelopment in this model (28). Aside from the tongue, numerous pharyngeal muscles contribute to the pharyngeal stage of swallowing, with motor innervation supplied by CN IX and X, both of which have been shown to have divergent development from normal in *LgDel* mice (7). Prolonged pharyngeal transit times correspond to impaired pharyngeal constriction (i.e., pharyngeal squeeze) by the tongue and pharyngeal muscles during swallowing, which is associated with increased laryngeal penetration of liquids and aspiration pneumonia risk in dysphagic patients (29–31). Importantly, CN IX and X also provide *sensory* innervation to pharynx and larynx. Clinical evaluation of laryngeal sensory function entails delivering puffs of air to the laryngeal mucosa to evoke the laryngeal adductor reflex (LAR or glottic closure reflex). A normal response is abrupt, brief (< 1 s) adduction of the vocal folds to protect the airway (19, 32). Our finding of an absent LAR in one *LgDel* mouse suggests that laryngeal sensory impairment may exist in some cases; however, testing with a larger sample size is needed to rule out effects from anesthesia, which is essential for performing LAR testing in mice. Importantly, none of the mice in this study demonstrated laryngeal penetration or aspiration while voluntarily drinking and eating during videofluoroscopic testing. This finding was not unexpected, as the larynx in mice resides in the nasopharynx (similar to human infants), which inherently protects the larynx from the path of the bolus (13–15). Regardless, *LgDel* mice display other deficits in feeding and swallowing that can serve as robust outcome measures in future preclinical therapeutic studies with this model.

In addition to CN IX, X, and XII deficits, CN V develops anomalously in *LgDel* mice (7). CN V provides motor innervation to the muscles involved in opening and closing of the jaw during drinking (licking) and eating (mastication). Although lick rate and rhythm were impaired in *LgDel* mice, the velocity of jaw open/close motion during drinking was indistinguishable from controls. Further, the mastication rate of *LgDel* mice during rotary chewing was no different from controls. However, alterations in jaw opening/closing velocity during chewing cannot be ruled out at this time, as this measure was not quantifiable using our JawTrack™ software. To answer this question, machine learning approaches are currently being incorporated into our software to permit automated detection and quantification of various masticatory patterns (e.g., biting, rotary chewing) in future work with this mouse model.

People with 22q11DS commonly have hypocalcemia due to parathyroid hypoplasia, and as a result, may experience paresthesias, tetany, muscle weakness, dysphagia, and fatigue (33). Therefore, it is important to note that while parathyroid hypoplasia has been established in the *LgDel* mice (34), calcium homeostasis has not been fully evaluated in this model. Although past studies and this study clearly demonstrate craniofacial and neurological origins of dysphagia, hypocalcemia could exacerbate the dysphagic deficits seen in the *LgDel* mice and therefore warrants further investigation.

### Variability in Oropharyngeal Anomalies in *LgDel* Mice Parallels That in 22q11DS

We found substantial, but in some cases variably, penetrant disruptions of several functional and anatomical measures of feeding and swallowing in adult *LgDel* mice. This variability accords with the variable penetrance of most 22q11 clinical phenotypes across individuals with 22q11DS, including variable penetrance and expressivity of features that may impact feeding and swallowing such as craniofacial abnormalities, congenital heart defects, and anomalies of the gastrointestinal tract (35). One limitation in the evaluation of craniofacial abnormalities associated with 22q11DS is the lack of established criteria for what is considered “normal” vs. “abnormal.” This is not helped by the fact that the identification of such anomalies is extremely subjective and limited by the quality of the photographs, radiographs, and recorded videos. Additional imaging and analytic approaches like those developed to assess cranial dysmorphology in mice with Down syndrome and other developmental disorders (36, 37) may be necessary to resolve this issue with appropriate quantitative and statistical precision. Though some progress has been made on digital diagnosis of 22q11DS (38).

### Incomplete Compensation for Developmental Disruptions Due to 22q11 Deletion

Both the frequency and the duration of eating and drinking were increased in adult *LgDel* mice, suggesting that these mice require more time and effort to ingest an equal amount of sustenance compared to WT littermates. Similarly, the *LgDel* mice groomed more frequently, but for the same cumulative duration as WT mice. This may signify that in order for the *LgDel* animals to achieve the same amount of grooming, they have to groom more frequently for shorter periods of time throughout the day, possibly due to fatigue and dysregulated tongue movement. These outcomes are supported by the decrease in lick rate observed in *LgDel* mice during VFSS with liquid consistency. Thus, with a diminished lick rate, the *LgDel* mice spend more time eating and/or drinking in order to achieve sufficient nourishment throughout the day and to maintain body weight. Although the oromotor deficits detected via HomeCageScan testing of only male mice appear to be more pronounced compared to the VFSS data obtained from male *and* female mice, we expect this “discrepancy” may be explained by differences in the types of behaviors assessed by each test rather than sex differences. HomeCageScan assessed the presence/absence of spontaneous oromotor behaviors over time whereas VFSS assessed characteristics of specific oromotor behaviors, specifically drinking and eating. However, we intend to investigate this hypothesis using a larger sample size of males and females in our future investigations with this model.

Additionally, other examined parameters unrelated to feeding and swallowing showed insignificant differences between the *LgDel* and WT mice as determined via HomeCageScan analysis, including walking slowly, coming down, and hanging vertically from a hang cuddled position (see Table 3 for definitions). Significant defects in the behaviors involving oromotor coordination, such as drinking, eating, and grooming coincide with the observed structural and functional issues within the *LgDel* mice. At the same time, the lack of significant differentiation in unrelated oromotor behaviors (walk slowly, come down, hang vertical from hang cuddled) indicate specificity of dysfunction in drinking and eating, as those actions that were not different in *LgDel* and WT mice do not involve known impairments associated with dysphagia and/or 22q11DS.

*LgDel* mice eat and drink more frequently and for longer durations. They may do so because of an underlying disruption of neural circuitry to execute the behavior or as a compensatory mechanism to minimize discomfort. Lack of coordination, slower execution (i.e., diminished lick rate) and fatigue would support the former possibility, particularly if cranial motor neurons are compromised or circuit integrity is altered (28). Moreover, altered nociceptive or mechanoreceptive innervation may also contribute to discomfort, thus supporting the latter mechanism. Finally, it is not unimaginable that slower nutrient intake over a longer duration may help the animals ingest food with fewer issues. This work suggests that feeding and swallowing difficulties observed in pediatric dysphagia are likely not fully resolved as the child develops further, leading to possible weight loss, food avoidance, aspiration, as well as frequent or chronic lung, naso-sinus or middle ear infections. Many of the oropharyngeal structural and cranial sensory-motor issues associated with dysphagia are due to underlying neurodevelopmental abnormalities; however, some of these difficulties may be ameliorated with slower nutrient intake. Similarly, fatigue involving suboptimal oropharyngeal structures or motor innervation for feeding and swallowing may be addressed by eating or drinking smaller amounts more frequently throughout the day. It would be an advantage in future work to use the same cohort of mice for VFSS, endoscopy, and behavioral assessments in order to investigate relationships between variables within the same mice.

It should be noted that mice are social animals by nature; therefore, it is possible that isolating mice from one another for 4 days during HomeCageScan testing may cause anxiety-related behaviors such as pacing or increased movement (39). However, such behaviors did not greatly vary between bins for individual animals, suggesting that anxiety level was not a confounding variable in this study. In addition, mice were rearing while eating and drinking during HomeCageScan testing, which was a necessary condition for automated detection of these behaviors; drinking and eating near the cage floor is too non-distinctive from other behaviors (e.g., grooming) for accurate quantitative video analysis. This rearing posture likely results in a more complicated task involving both oromotor and gross axial coordination and balance. This may be a confounding factor, given that children with 22q11DS are known to have marked neuromotor deficits affecting static and dynamic balance (40, 41) associated with diminished cerebellar volume (42). However, upon careful review of the HomeCageScan videos, there was no obvious evidence of balance or coordination deficits in either group of mice. It may be of interest in future work to investigate potential coordination and balance deficits and associated etiologies in this mouse model.

### Persistent Lung Inflammation in *LgDel* Mice

As pups, the *LgDel* mice showed evidence of aspiration and inflammation based on the presence of murine milk proteins, neutrophils, macrophages, and the accumulation of red blood cells within the lung tissue (7) The *LgDel* mice in this study also showed substantial lung inflammation, but it is not clear whether this was acute, chronic, or both. Thus, it is uncertain if the same degree of dysphagia seen in the *LgDel* pups, which appears to be acute during early life (6, 7), persists into adulthood or if the characteristics of feeding and swallowing difficulties change with growth, maturation, and behavioral compensation. People with 22q11DS can be immunocompromised, which may chronically impair their ability to clear aspiration-based infections (43). *LgDel* mice have not been evaluated immunologically, and they may also be immunocompromised to some degree, preventing them from adequately clearing aspirated milk and accompanying bacteria as pups. Like many other features of 22q11DS, the severity of immunological dysfunction is highly variable. Our finding that none of the mice in this study aspirated during videofluoroscopic testing may suggest that lung inflammation may be maintained from infancy rather than caused by ongoing aspiration during eating and drinking. In adults, however, aspiration may be more sporadic, thus not easily detected with a single episode of videofluorography. Thus, the lung inflammation may reflect a somewhat chronic state due to occasional aspiration events. In addition, videofluoroscopy lacks the visual resolution to permit detection of micro-aspiration associated with gastric reflux, which is the major pathogenetic mechanism of aspiration pneumonia (44). Typically developing mice cannot vomit or spontaneously reflux gastric contents, and therefore micro-aspiration is unlikely (45). Although unknown, it is possible that the major neurodevelopmental anomalies in *LgDel* mice may alter esophageal and gastric function, thus making gastric reflux and micro-aspiration possible. To address this knowledge gap, future studies should include histological assays of the lungs to detect the presence of proteins that are found in the adult mouse diet.

### Anomalous Feeding and Swallowing Throughout Life

The retention of feeding and swallowing deficits beyond the perinatal period in individuals with syndromic or non-syndromic neurodevelopmental disorders has not been considered thoroughly. We suggest that these sometimes subtle, but nevertheless significant difficulties in managing food intake and deglutition may establish subclinical challenges or clinical signs of diminished nutrition and weight regulation and increased ongoing aspiration-related naso-sinus or respiratory infections throughout the lifespan. Further, individuals with 22q11DS may be more vulnerable to age-related feeding difficulties or in extreme cases, oropharyngeal dysphagia due to early onset Parkinson's disease for which 22q11DS is a genetic risk factor (46, 47). Finally, additional neurological complications like traumatic brain injury or stroke—causes for acute dysphagia after a lifetime of optimal feeding in non-syndromic individuals—may occur with similar, or even enhanced frequency in individuals with 22q11DS vs. typical adults, and exacerbate chronic, sub-clinical feeding and swallowing difficulties.

The relationship between perinatal dysphagia due to 22q11 deletion and continued oropharyngeal dysfunction and feeding and swallowing difficulties may extend to other syndromic and non-syndromic neurodevelopmental disorders. Indeed, later arising issues with food avoidance, food preferences, and diminished or disordered food intake in clinically diagnosed disorders like autistic spectrum disorder or attention deficit-hyperactivity disorder may reflect undiagnosed perinatal feeding difficulties that are never fully corrected, due either to lack of intervention during a critical period or the degree of developmental disruption that established anomalies in oropharyngeal and craniofacial structures as well as neural circuits critical for feeding and swallowing. Thus, additional attention to issues of oropharyngeal competence and related behaviors should be considered more carefully in the management of a broad range of neurodevelopmental disorders throughout the lifespan.

Our results allow us to begin to understand how the severity of this neurodevelopmental disease may change with compensation in maturity, both behaviorally and biologically. Our findings in mice suggest there may be slight improvements observed over time in individuals with 22q11DS. Nevertheless, it appears that the majority of the deficits that occur during development are either stable or not fully corrected. Significant oropharyngeal motor disruptions and continued evidence of partially penetrant craniofacial anomalies most likely are due to early hindbrain and craniofacial patterning disruption, which cannot be effectively or fully corrected by developmental or post-natal compensatory mechanisms. Lung inflammation, which may be persistent, or acute and recurring due to occasional aspiration, is a less definitive, although suggestive, observation. Abnormalities in lick rate/rhythm and pharyngeal transit time suggest that the consequences of pathological cranial nerve or brainstem development remain unresolved as the animals mature. Further, the increased frequency and duration at which the *LgDel* animals spent eating and drinking corroborates this supposition. This work has therefore provided a deeper understanding of developmental to behavioral dimensions of dysphagia associated with 22q11DS, and provides a foundation for future work to identify effective therapeutic interventions.

## Data Availability Statement

The datasets generated for this study are available on request to the corresponding author.

## Ethics Statement

This animal study was reviewed and approved by the Institutional Animal Care and Use Committee (IACUC) at the University of Missouri – Columbia and The George Washington University.

## Author Contributions

LW and TL performed and analyzed fluoroscopic and endoscopic assessments, facial photographs, and skull radiographs, and performed post-mortem tissue collection. TL performed statistical analysis of the fluoroscopic and endoscopic data. HC conducted and analyzed all HomeCageScan behavioral data and performed the statistical analysis. GY imaged and analyzed the lung samples for evidence of inflammation. AH, FB, and TL developed and applied the JawTrack™ and VFtrack™ software to the fluoroscopic and endoscopic videos collected during this study. IZ contributed to the conception and design of the study. DM contributed to the conception and design of the behavioral aspects of this study. LW, HC, IZ, A-SL, and TL drafted the initial manuscript, figures, and legends. All authors contributed to manuscript revision and approved the submitted version.

### Conflict of Interest

The authors declare that the research was conducted in the absence of any commercial or financial relationships that could be construed as a potential conflict of interest.
